# Supplementary material for: The Major Factors Causing the Microspore Abortion of Genic Male Sterile Mutant NWMS1 in Wheat (Triticum aestivum L.)
Source: Int J Mol Sci. 2019 Dec 11;20(24):6252. doi: 10.3390/ijms20246252 (PMC6940770; doi:10.3390/ijms20246252)
Supplement: Supplementary file 1 [file ijms-20-06252-s001.zip › Supplementary Files/Supplemental Figures.doc]

**Figure S1.** Functional classification of the differentially expressed genes (DEGs) referring to the Gene Ontology (GO) database

**Figure S2.** Differentially expressed genes (DEGs) involved top 20 enriched pathways of the nine *K*-means clusters referring to KEGG database

**Figure S3.** The map of starch and sucrose metabolism pathway between *NWMS1* and WT at S1 stage referring to KEGG database ([https://www.kegg.jp](https://www.kegg.jp/)). Red: DEGs highly expressed in *NWMS1*; green: DEGs lowly expressed in WT; blue: DEGs highly expressed in *NWMS1* or WT; white: Genes expressed equally between *NWMS1* and WT.

**Figure S4.** The map of starch and sucrose metabolism pathway between *NWMS1* and WT at S2 stage referring to KEGG database ([https://www.kegg.jp](https://www.kegg.jp/)). Red: DEGs highly expressed in *NWMS1*; green: DEGs lowly expressed in WT; blue: DEGs highly expressed in *NWMS1* or WT; white: Genes expressed equally between *NWMS1* and WT.

**Figure S5.** The map of starch and sucrose metabolism pathway between *NWMS1* and WT at S3 stage referring to KEGG database ([https://www.kegg.jp](https://www.kegg.jp/)). Red: DEGs highly expressed in *NWMS1*; green: DEGs lowly expressed in WT; blue: DEGs highly expressed in *NWMS1* or WT; white: Genes expressed equally between *NWMS1* and WT.
